# Luminal B subtype: A key factor for the worse prognosis of young breast cancer patients in China

**DOI:** 10.1186/s12885-015-1207-z

**Published:** 2015-03-29

**Authors:** Li-Chen Tang, Xi Jin, Hai-Yuan Yang, Min He, Helena Chang, Zhi-Ming Shao, Gen-Hong Di

**Affiliations:** 1 Department of Breast Surgery, Fudan University Shanghai Cancer Center; Department of Oncology, Shanghai Medical College, Fudan University, Shanghai, 200032 China; 2 Department of Surgery, Revlon/UCLA Breast Canter, David Geffen School of Medicine, Los Angeles, CA USA

**Keywords:** Breast cancer, Young age, Subtype, Characteristics, Survival

## Abstract

**Background:**

The prognoses of young breast cancer patients are poor. The purpose of this study is to evaluate the different characteristics and prognoses among different subtypes of young breast cancer patients.

**Methods:**

The study included 1360 patients <40 years-old (y) and 3110 patients 40-50y with operable breast cancer in Shanghai Cancer Center, Fudan University. The characteristics, overall survival (OS) and disease-free survival (DFS) were compared.

**Results:**

The median follow-up was 54.1 months. More grade III tumors and more lymph-vascular invasions (P < 0.01) were presented in <40y group when compared with 40-50y group. More patients <40y presented with Luminal B (25.3% vs. 17.5%, P < 0.01) and triple negative (16.7% vs. 13.4%, P < 0.05) breast cancer while fewer had Luminal A tumor (48.5% vs. 59.2%, P < 0.01). Younger patients with tumors of both Luminal A and Luminal B types were at increased risk for worse DFS (P = 0.03, HR = 1.69, 95% CI = 1.05-2.72; P < 0.01, HR = 3.61, 95% CI = 2.50-5.22) when compared with the older patients. Patients <40y with Luminal B tumor had a two point five fold higher risk of death compared with older counterparts (P < 0.01, HR = 2.54, 95% CI = 1.35-4.79), however, a worse overall survival rate was not observed in the younger women with Luminal A breast cancer (P > 0.05). In multivariate analysis, Luminal B subtype was also a strong predictor of disease relapse (HR = 1.09, 95% CI = 1.01 to 1.19, P < 0.01) in younger patients with Luminal subtype tumors.

**Conclusion:**

Characteristics of breast cancer suggested a more aggressive biology in Chinese patients with breast cancer diagnosed at young age. Luminal B subtype may have a negative effect on the prognosis of young patients in China which should be validated further.

## Background

Breast cancer is the most common cancer in women in developed countries. According to the American Cancer Society, there will be over 200,000 new breast cancer patients in the USA in 2010, accounting for 28% of all malignant tumors in women [1]. It was estimated that only 11% of breast cancer patients are diagnosed between 35-44 years of age (y); and less than 5% are diagnosed under the age of 35 [2]. However, the figure reported is significantly higher in Asian, especially in Chinese population (10-20% in total) [3,4]. Breast cancer, although rare in young women, in known to have more aggressive biological characteristics and is associated with a more unfavorable prognosis compared with older women [4-6]. As previous reported, it is known that 5-year overall survival (OS) of young breast cancer patients was 80-90% and 5-year disease-free survival (DFS) was 70-80%, which were significantly lower than their older counterparts [7].

However, not all young breast cancer patients suffered from poor prognosis. To date, few well-designed study have focused on young age breast cancer and breast cancer subtypes. Although there is still no clear definition of ‘young’ breast cancer patients (ranging from 30 y to 45 y), the relationship of young breast cancer patients and breast cancer subtype remain great interest for investigation. The purpose of this study is to evaluate the different characteristics and prognoses among different subtypes of breast cancer between young breast cancer patients aged 39 or younger and the patients aged 40-50.

## Methods

### Patients and follow-up

**Figure 1:**
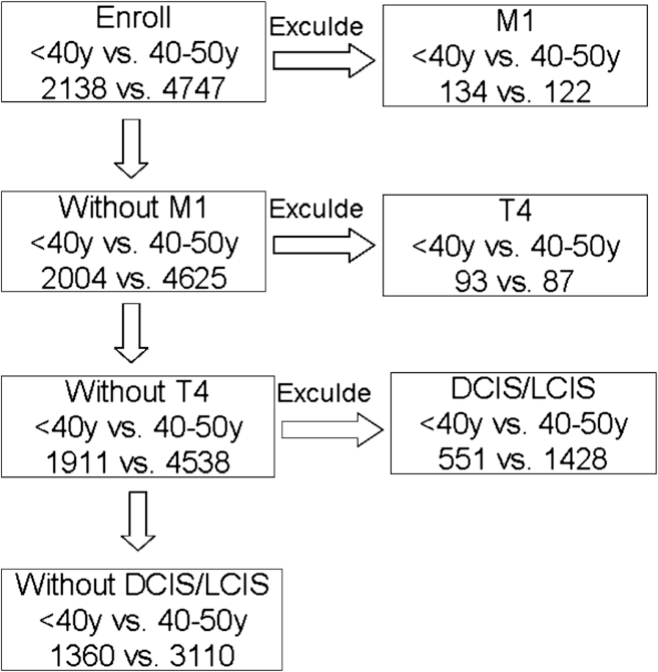
**CONSORT diagram of inclusion criteria and exclusion criteria for study.**

The medical records of 2138 and 4747 female patients aged <40y and 40-50y who had breast cancer diagnosed first time and were treated with breast surgery between Jan 1st, 2003 and December 31st, 2012, were retrospectively reviewed. This study was approved by the Ethics Committee of Shanghai Cancer Hospital, Fudan University. The information was retrieved from the database of the Shanghai Cancer Center, Fudan University, Shanghai, China. All patients were staged according to the 2007 American Joint Committee on Cancer (AJCC) guidelines [8,9]. Seven hundred and seventy-eight and 1637 patients were excluded from the two groups respectively according to the inclusion and exclusion criteria listed in Figure 1. Patients who required neoadjuvant therapy or who had metastastic disease were not included. Clinical and pathologic characteristics of these patients were classified by the inpatients’ computerised database, as required by the REMARK criteria [10,11]. All patients were required to have a complete physical examination, bilateral mammography, chest radioscopy, ECG, ultrasonography of breasts, axillary fossa, cervical parts, abdomen and pelvis, and routine blood and biochemical tests before surgery and adjuvant therapy when appropriate. Outpatient department records and records of personal contact with the patients, including routine correspondence and/or telephone calls, were used to follow the patients and determine the occurrence of loco-regional recurrence, distant metastasis or death. Patients returned to the Shanghai Cancer Center, Fudan University for follow-up every 3 months during the first 2 years, every 6 months in the third to fifth years, and annually after 5 years.

### Standard treatment

All patients were required to have a comprehensive physical examination and a pre-operative evaluation before surgery. All of the mastectomies and lumpectomies were R0 resection (margin-clear resection). Treatment with adjuvant chemotherapy, post-mastectomy radiotherapy and/or endocrine therapy was determined by the risk of relapse and was based on the standard of care at the time of surgery. Immunohistochemical (IHC) staining was used to evaluate the status of estrogen receptors (ER) and progesterone receptors (PR) in the tumors, as previously described. HER2 status was evaluated by IHC staining with (3+) being positive, (0 and 1+) being negative and (2+) requiring further evaluated by FISH. Four subtypes were defined: 1) Luminal A: ER(+) and/or PR(+), HER2(-), grade 1 or grade 2 tumors, 2) Luminal B: ER(+) and/or PR(+) and HER2(+) tumors or ER(+) and /or PR(+) and HER2(-) grade 3 tumors, 3) HER2+: ER(-), PR(-) and HER2(+) tumors, 4) Triple negative: ER-, PR-,HER2- tumors.

### Endpoints and statistical analysis

Overall survival (OS) was defined as the time from the first diagnosis of primary breast cancer to death from any cause. Disease-free survival (DFS) was defined as the time from the diagnosis of breast cancer to the development of any local recurrence or metastatic disease. Patients without any evidence of relapse or death were censored at the last date they were known to be alive.

Statistical analysis was carried out with SPSS, version 16.0 (SPSS Inc, Chicago, IL). The Chi-square test was used to compare patient and clinical-pathological characteristics between sub-groups, and the nonparametric Kruskal–Wallis rank test was used for ordinal categorical variables. Survival distributions were estimated by the Kaplan–Meier method, and the comparison between subgroups was done by the log-rank test. Multivariate analysis was carried out using Cox’s proportional hazard regression model, and hazard ratios (HR) are presented with their 95% confidence intervals. All statistical tests were two sided, and P < 0.05 was considered significant.

## Results and discussion

### Clinicopathologic characteristics

One thousand three hundred and sixty patients below the age of 40y and 3110 patients from 40y to 50y with primary breast cancer were included in the study. The patient characteristics of the two groups were summarized in Table 1. The median age was 35.2y and 46.0y respectively for the two comparison groups. Eighty patients (5.9%) younger than 40y had a family history in comparison with 1.4% in the group of 40-50y patients (P < 0.05). Two hundred and twenty-three (16.4%) patients aged 39 or younger received breast conservative surgery (BCS) while fewer older patients (7.6%) underwent BCS. A larger proportion of younger patients received chemotherapy (93.2% vs. 90.1%) and radiotherapy (25% vs. 17.4%). However, the difference were not statistically significant in all adjuvant therapies between the two groups (P > 0.05, Table 1).

**Table 1:** **Clinicopathologic characteristics of patients according to age**

Age	<40y		40 ~ 50y		P value
character	No.	%	No.	%	
T stage					<0.01
T1	520	38.2	1072	34.5	
T2	625	46.0	1914	56.3	
T3	215	15.8	124	9.2	
N stage					0.07
N0	626	46.0	1580	50.8	
N1	324	23.8	787	25.3	
N2	324	23.8	606	19.5	
N3	86	6.4	137	4.4	
Family history					<0.01
Yes	80	5.9	44	1.4	
No	1280	94.1	3066	98.6	
Tumor Grade					<0.01
Grade I	56	4.1	93	3.0	
Grade II	775	57	2233	71.8	
Grade III	529	38.9	784	25.2	
Tumor type					0.4
IDC	1216	89.4	2762	88.8	
ILC	60	4.4	168	5.4	
Others	84	6.2	180	5.8	
LVI					<0.01
Positive	413	30.4	790	25.4	
Negative	947	69.6	2320	74.6	
ER status					<0.01
Positive	821	60.4	2133	68.6	
Negative	539	39.6	977	31.4	
PR status					0.9
Positive	826	60.7	1894	60.9	
Negative	534	39.3	1216	39.1	
HER2 status					0.01
Positive	390	28.7	647	20.8	
Negative	970	71.3	2463	79.2	
Subtype					<0.01
Luminal A	660	48.5	1959	59.2	
Luminal B	344	25.3	579	17.5	
Her2 positive	129	9.5	327	9.9	
Triple negative	227	16.7	445	13.4	
Srugery					<0.01
BCS	223	16.4	236	7.6	
Mastectomy	1137	83.6	2874	92.4	
Endocrine therapy					0.7
No	204	15.0	442	14.2	
Yes	1156	85	2668	85.8	
Chemotherapy					0.4
No	92	6.8	308	9.9	
Yes	1268	93.2	2802	90.1	
Radiotherapy					0.1
No	1020	75.0	2568	82.6	
Yes	340	25.0	542	17.4	

*Abbreviation*:

*IDC* intraductal carcinoma.

*ILC* intralobular carcinoma.

*LVI*  lymphovascular invasion.

*ER* eostregen receptor.

*PR* progesterone receptor.

*BCS* breast conservative surgery.

Younger patients tended to have a higher T stage (P < 0.01) and the trend of a higher N stage (P = 0.07) in comparison with the group of older patients. More grade III tumors (38.9% vs. 25.2%, P < 0.01) and more lymph-vascular invasions (30.4% vs. 25.4%, P < 0.01) were presented in the <40y group when compared with 40-50y group.

Breast cancer in women younger than 40 had a lower ER positive rate (60.4% vs. 68.6%, P < 0.01) but a higher HER2 positive rate (28.7% vs. 20.8%, P < 0.01) between the pair. The PR positive rate was similar between the two groups (P = 0.9). More patients <40y presented Luminal B (25.3% vs. 17.5%, P < 0.01) and triple negative (16.7% vs. 13.4%, P < 0.05) breast cancer while less of them presented Luminal A tumor (48.5% vs. 59.2%, P < 0.01, Table 1). The percentage of patients with HER2 positive, hormone receptor negative breast cancer was similar in both groups (9.0% vs. 9.9%, P > 0.05, Table 1).

When the cut-off age was set at 35y, almost the same characteristics as women aged 39 or younger were found with more of these patients having family history of breast cancer and BCS. Similarly, tumor of patients <35y presented higher T stage, N stage, higher grade and more lymph-vascular invasions. The proportion of Luminal B and triple negative breast cancer in <35y patients was also preponderant over that in 35-50y patients (Data not shown).

When we analyzed the subtypes according to molecular classification, more patients with HER2+ and Triple negative breast cancer presented higher T and N stage in comparison with those with Luminal groups (P < 0.05). There was no significant difference in surgical modality or adjuvant therapy strategy among the four subtype groups (P > 0.05,data not shown).

### Survival analysis

**Figure 2:**
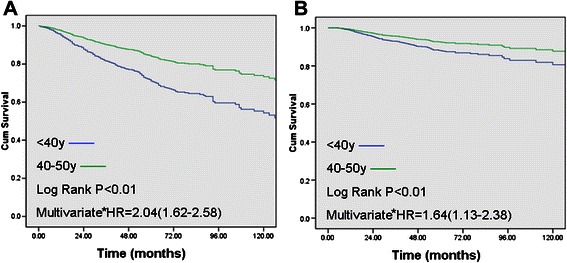
**Overall survival (A) and disease-free survival (B) of <40y group vs. 41-50y group.** *Adjusted for tumor size (≤5 cm v >5 cm) and lymph node status. HR, hazard ratio.

The median follow-up of the reported patients was 54.1 months (58.8 months for <40y group vs. 50.5 months for 40-51y group). The 5y DFS was 72% vs. 83% (P < 0.01) and the 5y OS was 87% vs. 93% (P < 0.01), in favour of patients in the 40-50y group (Figure 2A,B).

There were significant differences between the two age groups with regards to the ascending of T stage and N stage subgroups (P < 0.01 both for OS and DFS, data not shown). The survival curves of the <40y group fell more sharply as the tumor grade increased when compared with women of older age group (P < 0.01 both for OS and DFS, data not shown). The effect of age on survival was significantly different between <40y group and 40-50y group, adjusted for tumor size and/or lymph node status (P < 0.01, HR = 1.64, 95% CI = 1.13-2.38 for OS and P < 0.01, HR = 2.04, 95% CI = 1.62-2.58 for DFS, Figure 2).

**Figure 3:**
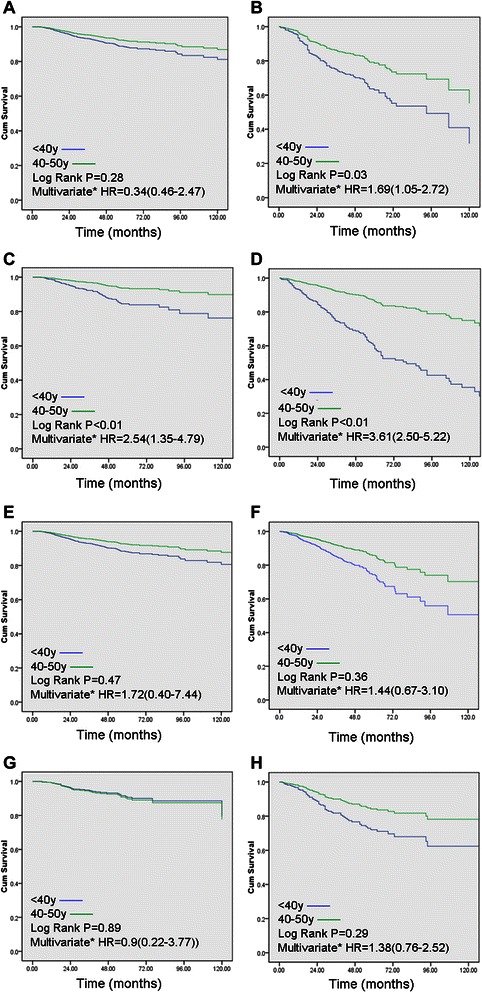
**Overall survival (A,C,E,G) and disease-free survival (B,D,F,H) between the two age groups, according to subtype (A/B: Luminal A, C/D: Luminal B, E/F: HER2, Triple negative: G/H).** *Adjusted for tumor size (≤5 cm v >5 cm) and lymph node status. HR, hazard ratio.

When compared different subtypes of breast cancer in the two different age groups (adjusted T and N stage in the analysis),younger patients with tumors classified as Luminal A and Luminal B type were at increased risk of poor DFS (P = 0.03, HR = 1.69, 95% CI = 1.05-2.72; P < 0.01, HR = 3.61, 95% CI = 2.50-5.22). Additionally, patients <40y with Luminal B tumor had a two point five fold higher risk of death compared with older counterpart (HR = 2.54, 95% CI = 1.35-4.79, Figure 3) while there was no significant risk of death associated with Luminal A subgroup (P > 0.05). In contrast, HER2+ and triple negative breast cancer showed no significant differences in DFS or OS between the younger and the older groups (P > 0.05, Figure 3). Furthermore, Luminal B subtype was a strong prognostic factor of disease relapse (HR = 1.09, 95% CI = 1.01 to 1.19, P < 0.01) in younger patients with Luminal subtype tumor.

Almost all the same trends were identified when comparing the <35y and ≥35y groups for subgroup comparisons as those found when comparing the two groups <40y and 40-50. Young age was shown being one of the major predictors for worse survival with respect to the larger tumour size group, the higher lymph node rate group and the Luminal B group.

### Discussion

Breast cancer is a rrare in young women [12,13]. Consensus has been reached that breast cancer in young age is different from those of less young age [14]. Young patients often present with more aggressive biologic behavior, such as advanced stage, less ER positive expression, higher histological grade and more lymph-vascular invasion [15].

To our acknowledgement, this is the only study focused on breast cancer subtype and survival with largest sample of young patients. The most common surgery was mastectomy with axillary lymph node dissection (83.6%) while breast conserving surgery was performed in only 16.4% patients. Studies have shown that young age alone is not a contraindication for breast conservation, but the risks should be discussed with the patient [16,17].

Our study has shown that young breast cancer patients are found at a more advanced stage and have more aggressive histologic and molecular characteristics, irrespective of the cut-off age at 35y or 40y. Younger patients frequently suffered from a higher T stage, a higher N stage andmore de-differentiation, which are all indicative of a poorer prognosis . In addition, this study is also consistent with the results of the Colleoni et al [12] that more vessel invasions and HER2 overexpresed tumors were more frequently found in breast cancer occurred in patients of young age. It was reported that approximately one-quarter of all breast cancer cases are hormone receptor negative, but a large proportion of hormone receptor negative tumor occurs in younger women [18]. Our study showed that younger patients had fewer hormone receptor-positive cancer (60%) in comparison with older women group aged between 40-50 (68%). These data are consistent with previous studies from other countries reporting less hormone receptor positivity in younger breast cancer patients [19].

Although environmental, lifestyle and genetic factors may have a great impact on the survival of young breast cancer patients, the TNM stage and biological features determine the prognosis. In our study, we found at a more advanced stage and have more aggressive histologic and molecular characteristics, irrespective of the cut-off age at 35y or 40y. Even in multivariate analysis, young age was a key point to both DFS and OS.

In this study, we found that Luminal A, Luminal B were the most common subtype in Chinese young breast cancer patients, followed by triple negative and HER2 positive with negative hormone receptor subtypes. Others reported that the Luminal B and triple negative were the most common subtypes in young patients because of the different definition of breast cancer subtype which associated with a increased risk of death in young patients [20]. In our study, young patients with Luminal B breast cancer had a 2.5 fold higher risk of death and 3.6 fold higher risk of relapse disease compared with the older counterpart Luminal A, HER2+ or triple negative, had similar survival outcomes in the two age groups. These findings were supported by recent studies reported by Chang’s group [21]. This finding has the accordance with the report of Lin et al [22]. Our study and others suggest that HER2 over-expression, high tumor grade and hormone receptor status may all be important prognostic factors in young breast cancer patients. Hormone receptor positivity may be important in managing young breast cancer patients because of the long exposure to the high level of estrogen [23]. Therefore, patients of young age with Luminal B tumor defined by positivity of both hormone receptors and HER2 expression will benefit from the combination of anti–HER2 target therapy, endocrine therapy and chemotherapy.

Our study also has some limitations in clearly defining young women breast cancer. Some of our cases had very limited follow-up which were censored too early, which might influence the survival analysis. Additionally, it is known that young breast cancer has a disproportionately high presentation of BRCA1 and BRCA2 mutations [24,25]. However, genetic testing has not been well established in China. Further genetic and genomic investigations may accelerate our understanding of breast cancer in young patients. Lastly, the definitions of subtypes of breast cancer varied in the literature which could mislead the analysis and therefore conclusions. More reliable classification is needed to better address the tumor biology in young women’s breast cancer.

## Conclusion

In conclusion, tumors in young women are more likely to present advanced disease with high grade and lymphovascular invasion. Young breast cancer patients in China are more likely to have Luminal B and triple negative tumor. Young patients with Luminal B tumor, but not other subtype, had the highest risk for relapse and death events over the older patients. Further research is required to investigate new treatment strategies in young patients with breast cancer, especially Luminal B subtype.

## Abbreviations

Y: Years-old
OS: Overall survival
DFS: Disease-free survival
CI: Confidence intervals
IDC: Intraductal carcinoma
ILC: Intralobular carcinoma
LVI: Lymphovascular invasion
ER: Eostregen receptor
PR: Progesterone receptor
BCS: Breast conservative surgery
